# School Memories of Preservice Teachers: An Analysis of Their Role in the Conceptions About the Relationships Between Emotions and Teaching/Learning Processes

**DOI:** 10.3389/fpsyg.2021.690941

**Published:** 2021-10-13

**Authors:** Rodolfo Bächler, Romina Salas

**Affiliations:** ^1^Escuela de Psicología, Facultad de Ciencias, Universidad Mayor, Las Condes, Chile; ^2^Campus San Felipe Chile, Universidad de Playa Ancha, Valparaiso, Chili

**Keywords:** emotions, teaching, learning, beliefs, imagery, preservice teacher, school memories

## Abstract

With the aim of exploring the role of emotional elementary school memories that preservice teachers have, in their conceptions about the relationships between emotions and teaching-learning processes, a study was carried out. The sample consisted of all first-year preservice teacher from one campus of a Chilean public university (167 students). The study used a mixed method research composed of two stages. In the first part (quantitative), the conceptions of the preservice teachers were evaluated by means a dilemma questionnaire. In the second part (qualitative), a retrospective imagery was applied to a subgroup of thirty participants from the total sample with the aim of detecting and analyzing emotionally significant memories of teaching-learning situations in elementary school. The result of the first part of the study showed that most of preservice teachers maintain conceptions that separates affect and cognition as non-integrated processes. The results of the second part revealed that preservice teachers remember different types of experiences, most of them are associated with the characteristic and behavior from their primary school teachers. Finally, the recovered memories were analyzed in terms of their relationship with conceptions, concluding that having experienced fear in school could be a factor related to the type of conceptions held.

## Introduction

A considerable number of studies in recent years have highlighted the role of emotions on human interaction (Bächler and Poblete, 2012) and on teaching practice (Atkinson, 2002; Sutton and Wheatley, 2003; Pekrun, 2005; Chang, 2009; Fried et al., 2015; Frenzel et al., 2016). Because of this, some authors says that now, in the world exists an emotional gyro in education (Dernikos et al., 2020). In this context a particularly important question to respond, is to know how teachers conceive the role of emotion in educational processes.

### Teachers’ Conceptions of the Relationships Between Emotions and Teaching and Learning Processes

Recently, teachers’ Conceptions about the relationships between Emotions and Teaching and Learning processes (CE-TL) have been investigated (Bächler et al., 2018). CE-TL refers to teachers’ conceptions about the role that emotions play in learning verbal content such as math, language, or others of the same type. In this context, conceptions would be understanding in a similar way to Dejene (2020) who define this term as beliefs that “guide a teacher’s perception of a situation and will shape actions” (p. 3). Following Dejene definition of conceptions, CE-TL influence the pedagogical acts that teachers execute in the classroom in terms of integrate or not the emotions in the teaching and learning process. This is an especially important question, because neuroscientist discovered the deeply relation existing between emotions and reasoning in our mental life (Gu et al., 2012; Pessoa, 2013). As Candiotto (2019) says “emotions are now understood as a constitutive element of human rationality, grounding concept creation and deliberative thinking, and partaking in the various cognitive processes, rather than being framed in opposition to rationality” (p. 4). From this point of view, moreover, emotions can be conceived as complex phenomena who are composed by phenomenological and representational properties that are integrated in a complex gestalt that people name “emotions.” Constructionist approaches (Barrett and Russell, 2015) assert that emotions result from the integration of core affective states and conceptual categorization processes that lend meaning to the experience and are the basic “building blocks” of every emotion (Barrett et al., 2007; Bächler and Pozo, 2016).

**FIGURE 1:**
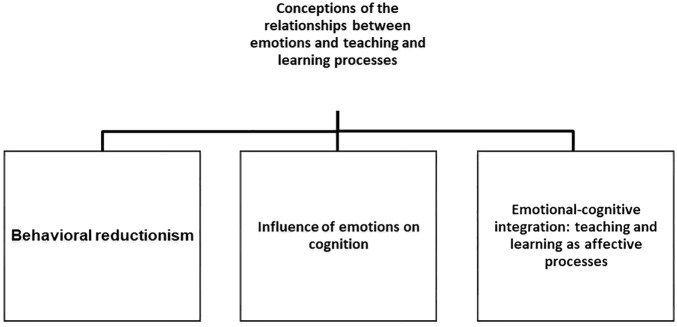
Conceptions about the relationships between emotions and teaching and learning processes.

Back to the research of conceptions about the relationships between emotions and teaching and learning processes the specialized literacy identify that CE-TL are organized in three conceptions as is shown in the Figure 1 and described below:

### Behavioral Reductionism: The Absence of Emotions in Teaching-Learning

The first conception, called “behavioral reductionism,” is a perspective characterized by an inadequate understanding of affections as mental states and therefore assimilates emotions to their associated behaviors. Teachers who hold this perspective act in a similar way to what happens with children, believing that being sad is crying and being happy is laughing (Pons et al., 2004), without establishing relationships between affections and the teaching-learning process. This conception is characterized by the motto: “absence of emotion in teaching-learning.”

### Influence of Emotions on Cognition: Emotions as Context of the Teaching-Learning Process

The second perspective, called “Influence of emotions on cognition,” consists of a conception from which the subjective and qualitative character of emotions is understood. Teachers who participate in this approach can identify a role for affections on all types of learning, including those that deal with verbal and symbolic curricular content, such as mathematics or chemistry. From this perspective, relationships between emotions and the educational process are reduced to the valence of the former, in a manner like the point of view maintained by the psychological current known as “positive psychology” (Stout, 2000). Under this approach, if an emotion is pleasant, then it will always be a favorable element of learning. On the contrary, if the experience of an affection is unpleasant, then it is conceived as an obstacle to teaching and learning.

### Emotional-Cognitive Integration: Teaching and Learning as Affective Processes

Beyond the specific differences between the two conceptions described above, both share the fact of being supported by a dualism between emotions and processes classically considered cognitive. This is a characteristic that it changes in the third conception identified, called “Emotional-cognitive integration,” defined by considering emotions as the center of teaching-learning process. This conception does not establish substantive ontological differences between affective and cognitive processes that are consistent with a series of findings in the field of neurosciences (Duncan and Barrett, 2007; Damasio, 2010; Gu et al., 2012; Pessoa, 2013). From this point of view, teaching and learning are understood as processes that start under an emotional format, which then mutate into symbolic representations. This perspective implies a more flexible view regarding the valence of emotions, from which it is understood that learning can be facilitated by emotions experienced as unpleasant (negative valence), as well as can occur, that emotions that feel pleasant (positive valence) can hinder this process.

Finally, to complete the knowledge about CE-TL, it is necessary to comment that the perspectives described above, served as a framework for a larger study with a sample of 470 teachers of different profiles, who were evaluated with respect to their conceptions with a dilemma questionnaire (Bächler and Pozo, 2020). Results of this study gave a generalized orientation, in the sample of teachers, in the sense of considering emotions under the mode called “Influence of emotions on cognition.” In addition, this study allowed us to verify that teachers mostly conceive affections as states that influence teaching-learning processes depending on their valence: if we feel good, we learn more; if we dislike an experience, we learn less. On the other hand, the group of teachers who expressed a more complex conception about the role of emotions in educational processes (Emotional-Cognitive Integration), turned out to be the smallest group of all those identified. These results lead to the question of how teachers’ conceptions develop, a problem who is analyzed in the following section.

### Development of the Conceptions: Importance of the School History

Understanding the development process of the conceptions is a topic of special interest to improve educational systems, among other reasons, since there is a relationship between conceptions and pedagogical practices (Buehl and Beck, 2015). In addition, conceptions held by preservice teachers^1^ acts as a filter to which they perceive new knowledge and experiences during their university career (Fives and Gill, 2015). Thus, if we can better understand the origin of these students’ conceptions, adjustments can be made to the way in which teacher-educators work to collaborate with information that can deliver more specific comments to support initial teachers training (ITT). Thus, from a constructivist approach, not only the information is important that is delivered to future teachers through the ITT, but it is also important to consider the conceptions that student teachers have about the different facets of the educational process. This is because all new knowledge acquired during training is built on interaction with those conceptions. In this context, quality ITT should contemplate the conceptions that preservice teachers “bring” into the teacher training process. Therefore, it is necessary for teacher educators to know what these conceptions are and what characteristics they possess (Murphy et al., 2004).

Buehl and Fives (2009) identify six sources of teacher conception formation: formal education, observational learning, formal knowledge bodies, collaboration with others, personal teaching experiences and self-reflection. A quick analysis of these factors reveals that the first two, formal education and observational learning, can originate before ITT. Similarly, several authors affirm that experiences in the school system of future teachers constitute a factor that models their conceptions about education (Rokeach, 1968; Lortie, 1975; Stuart and Thurlow, 2000; Bächler, 2017). These are vivid images that future educators have about pedagogical work, which are acquired during their schooling, and that will influence their interpretations of what happens in the classroom during their practice as teachers (Leal, 2005).

It is not, that preservice teachers consciously and methodically imitate their own instructors. Instead, future teachers internalize implicit teaching models generated from their own experience as apprentices in the school system by observing the actions of their teachers. These episodic memories are stored and provide future teachers with an accessible repertoire of behaviors for use in the classroom (Nespor, 1987; Bächler, 2017). Miller and Shifflet (2016) refers to these memories as:

“ghosts that continue to shape and haunt the conversations and interactions of adults in the school context. The term ghost is used as a symbol for school memories that follow individuals into new stages of their lives; as shadows that continue to inform one’s thoughts and behaviors.”

The terms “ghost” and “shadow” suggests that they are not explicit representations, like ideas or theories about the educational process. We can understand these memories as implicit representations about learning and teaching. However, the question is: “what characteristics do these memories or implicit representations have?”

### Characteristics of Memories as Implicit Representations

A trait that appears repeatedly in the literature that analyzes the characteristics of implicit representations is its iconic character (Calderhead and Robson, 1991; Ballesteros, 1992; Pajares, 1992). This cognitive function is characteristically human, as demonstrated by evidence from different approaches and techniques (Finke et al., 1992; Singer, 2008). At the educational context, these images function as filters for new information obtained and act as “intuitive scripts” during the teaching process (Goodman, 1988). This becomes especially relevant in those moments when teachers face situations that are not possible to address through explicit and verbally coded planning. Sometimes, these kinds of properties can take the form of an “iconic teacher,” who represent a desired self for the student teachers (Miller and Shifflet, 2016).

In another hand, some authors describe implicit representation as “experiential processing;” and, in addition to being related to images, they have emotional properties that guide behavior from their connections to past events (Epstein and Rosemary, 2000/2001). According to Atkinson (2002), both vision and intuition represent alternative ways of knowing that differ from reasoned analysis and are based on the ability to infer truths from prior knowledge, without conscious articulation. This suggests that in addition to their iconic character, these representations are stored in the form of experienced sensations resulting from the association of recurring situations or that have similar features, in a manner which has been called “somatic markers” (Damasio, 2001).

Characteristics of the implicit representations discussed above allow us to understand that any methodology that aims to know the memories or implicit representations that the preservice teachers have formed during their passage through the school system, will imply the use techniques that permit awareness of the stored images and emotions (Bächler, 2017).

Considering the previous analysis, the following research questions arise: Which are the preservice teachers’ conceptions regarding relationships between emotions and the teaching-learning processes? Which emotional characteristics have the primary school memories from the future teachers? How will these memories relate to CE-TL that preservice teachers have?

## Materials and Methods

This study uses a mixed method research (MMR) who integrate quantitative and qualitative techniques, a kind of methodology research which provides a better understanding of the research problems than using qualitative and quantitative techniques in a separating way (Creswell, 2007). As Johnson et al. (2007) say, MMR is “the type of research in which a researcher or team of researchers combines elements of qualitative and quantitative research approaches (e.g., use of qualitative and quantitative viewpoints, data collection, analysis, inference techniques) for the broad purposes of breadth and depth of understanding and corroboration” (p. 123). The latter is a very important characteristic to consider if one takes into account that a central objective of the present study is to understand how the emotionally significant school memories of future teachers contribute to the development of their conceptions about the role of emotions in educational processes (CE-TL). The achievement of this objective implies a work in two stages of investigation, each one with specific characteristics. First, the use of a methodology is required to identify the CE-LT of future teachers to have a diagnosis of the perspectives they have on this topic (quantitative component). Second, a process of exploration of the subjective world of the participants must be carried out (Flick, 2007); and more specifically, the detection and analysis of emotionally significant memories about teaching-learning situations in primary school (qualitative component). Finally, it is necessary to advance through the analysis, toward an integration of the two types of data obtained in the sense of describing some type of relationship between them. This task connects with the essence of mixed methods studies according to Anguera et al. (2020), which is, that “they contain qualitative and quantitative components that must be integrated to ensure the mixing of the information they carry” (p. 7). Considering the above, the present study can be defined, according to the typology established by Leech and Onwuegbuzie (2009) as a partially mixed sequential equal status design (P3), a “study with two phases that occur sequentially, with the quantitative and qualitative phases having equal weight” (p. 270).

### Participants

All first-year teacher students from one campus of a Chilean public university, were invited to participate in the study. The invitation was made during an induction workshop to university life, and all the students accepted to participate (167 student teachers). The average age of the sample was 19 years. 74% of the students were female, 20% male, and 6% identified with other types of gender. 74.9% declared that they had no previous university studies, while 22.8% indicated that they had incomplete university previous studies. 24% of the participants came from a public school, 50.6% came from a public private school, and the rest, corresponding to 25.4% of the sample, came from a private primary school. In the first part of the study (quantitative) the total of the sample participates. In the second part (qualitative) a subsample of thirty students’ teacher was selected to participate. As is detailed in the section “Data Production Techniques,” this subsample was formed by two groups of fifteen students, each associated with a particular CE-TL.

All students voluntarily participated in the study. Anonymous use of the collected data was guaranteed by signing an informed consent protocol.

### Data Production Techniques

In the present study, two types of instruments were used, each associated with different stages of the research process. For the initial phase of the study, which consisted of the evaluation of CE-TL, we used a questionnaire of previously validated dilemmas (Bächler and Pozo, 2020), which was applied to the entire sample of students. The instrument contained twelve teaching-learning situations, which posed an educational conflict to which there were three response alternatives. Each response option was associated with one of the following conceptions about CE-TL described in previous sections. The following sample item gives an idea of the characteristics of the instrument:

Robert has had some difficulties learning mathematics and lately, he has been working to improve their performance. The teacher who has been attentive to his efforts decides to bring him to the board to solve a problem. However, Robert is not able to solve the problem, showing sadness by the situation. So, the teacher

a. Corrects Robert’s mistake by teaching the correct procedure to solve the exercise and try to reassure him by telling him that learning mathematics is a slow process that must be done step by step.
b. Encourages Robert by indicating him that his failure does not tarnish the effort and progress he has shown and urges him to continue working until meeting the goals he has set for himself.
c. Allows Robert to express his sadness and then invites him to discuss how it relates to their difficulties in learning mathematics.

Subsequently, in a second part of the investigation, consisting of the identification, description, and analysis of the emotionally significant memories of the preservice teachers, the use of imagery was employed. This is a technique from clinical psychology used in other theoretical contexts (Hanrahan and Vergeer, 2000/2001; Kealy and Arbuthnott, 2003). This technique was chosen because it allowed a more direct access, with a minimum of linguistic reconstruction of the memories.

Design and testing of the imagery contemplated two stages aimed at refining the technique until obtaining a finished proposal. A first version was developed in collaboration with a clinical psychologist, an expert in this type of work. Then, with a theoretical design of the technique and a prepared script, an initial test was carried out with eleven teachers who were invited to participate in a retroactive visualization aimed at rescuing memories of their school biography. Teachers were later interviewed regarding their experience during the activity, as well as about the characteristics of the applied technique. The feedback collected allowed the imagery to be refined, defining the steps to follow to improve the technique. Considering the conceptual foundations of the study, it was decided to focus the imagery on the recovery of memories associated with primary education, which occurred in the school classroom and during learning of verbal content. With a new version of the instrument, a second application was made with a group of eight preservice teachers. The results of this application refined the last details of the instrument, especially with regards to the contextual conditions required for its use with adolescents, thus achieving its definitive design.

Finally, with the imagery already elaborated, the instrument was applied to a subsample of thirty preservice teachers. This subsample was formed by two groups of fifteen students, each associated with a particular CE-TL. The first group corresponded to students who had, in accordance with the results obtained in the questionnaire of dilemmas applied during the first part of the study, a position close to the most basic conception: “Behavioral reductionism.” The other selected group was closer to the more complex conception, called “emotional-cognitive integration.” The division into two groups that had opposite CE-TL, aimed to facilitate a clearer contrast between the memories detected, based on their relationship with the type of conception maintained.

The imaginary was applied in six sessions, with five students in each session. Once the imagery was completed, all participants received numbered booklets and pencils to freely describe their recovered memories.

### Data Analysis

In the first part of the study, with the objective of determining the conceptions that were preferred and those that the students rejected, a contrast of means of the answers from the dilemma questionnaire was made, using the student’s *t*-test for related samples. At the same time, with the objective of determining the existence of different perspectives on the role of emotions in teaching-learning processes, a cluster analysis of K means was carried out. This last analysis allowed the differentiation between the two groups of fifteen students that formed the subsample to which the imagery was subsequently applied.

**FIGURE 2:**
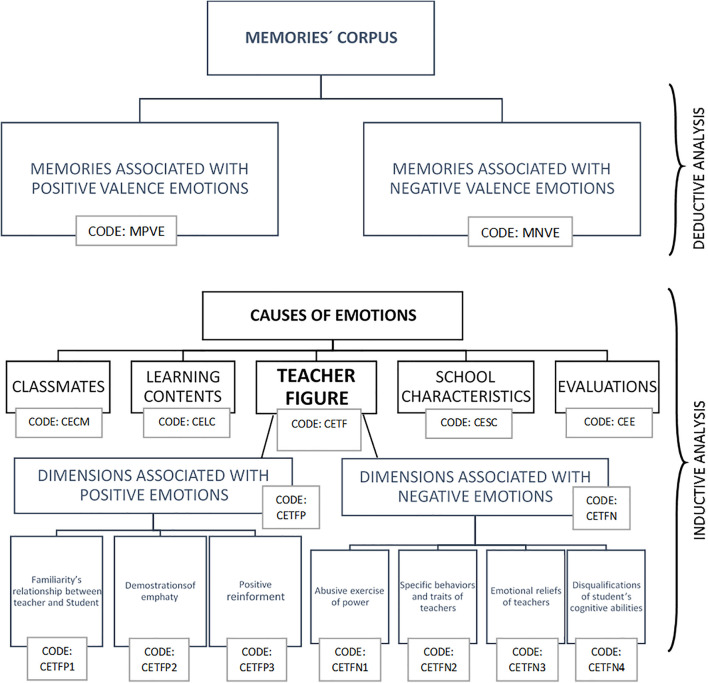
Coding and categorization process.

Regarding the second part of the study, corresponding to the results obtained by applying the imagery, the memories recovered by each student were transcribed and analyzed using grounded theory (Flores, 2013) using Atlas ti. To carry out the qualitative analysis, first, a deductive categorization was made, coding the memories according to their emotional valence, given the importance of this category to distinguish between different types of emotions (Charland, 2005). In a second part, an inductive analysis was carried out with the objective to identify the causes of the emotional memories referred from the students. Finally, in a third stage, a “zoom” to the “Teacher” category was made, trying to identify different aspects of the teacher figure, who are associated with positive and negative emotions. Also, non-parametric statistical procedures were carried out to complement this last analysis. The Figure 2 summarize the process of categorization and coding the qualitative data.

Once the previous steps have been carried out, corresponding, on one hand, to the evaluation of the conceptions held by the Student Teachers (CE-TL), and on the other, to the recovery of school memories, we sought to detect the existence of possible relationships between this to aspects of the study. To reach this goal, a means contrast was done using the student’s *t*-test for independent samples.

## Results

Results are presented as follows: First, the findings related to the initial part of the study, consisting of the evaluation of the CE-TL through the dilemma questionnaire, are presented. Then, in a second section, results obtained from the application of the imagery are presented, consisting of the identification, description, and analysis of the emotional memories of the preservice teachers. Finally, in a third section, an analysis is made of the possible CE-TL existing differences evaluated during the first stage, according to the emotional memories identified during the second stage of the study.

### First Part Results: Evaluation of the Conceptions About Emotions and Their Relationship With the Teaching/Learning Processes That Preservice Teachers Have

#### The Conceptions Preferred and Rejected by Future Teachers

**FIGURE 3:**
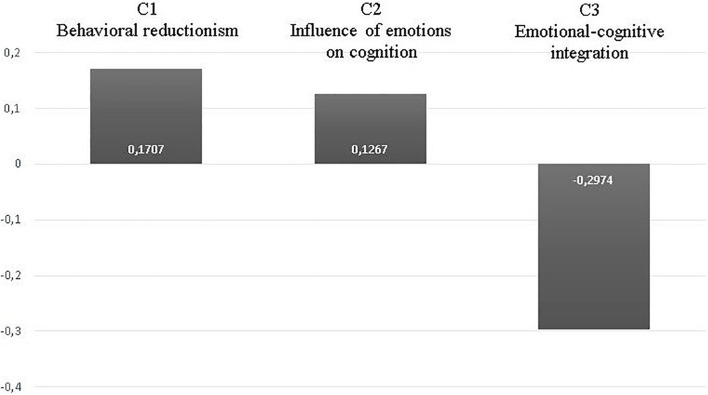
General response trends.

One of the study questions was to investigate “Which are the conceptions of future teachers about the relationships between emotions and teaching-learning processes.” To answer this question, we carry out an analysis consisting of determining the preferred and rejected conceptions by the students. Figure 3 shows the results of this analysis.

When analyzing the general tendencies of the students’ responses, there was an acceptance mostly shared by the concepts “Behavioral reductionism” and “Influence of emotions on cognition.” Conversely, there was a wide rejection of the more sophisticated conception “Cognitive emotional integration.” This situation is observed both when comparing the averages of response for said conception against “Behavioral reductionism” [(Student’s *T* for related samples: *t*(166) = 10,684, *p* = 0.001, *r* = −0.674)], as well as when contrasted with the conception “Influence of emotions on cognition” [(Student’s *T* for related samples: *t* (166) = 13,447, *p* = 0.001, *r* = −0.177)]

#### Profiles of Conceptions About the Relationships Between Emotions and the Teaching-Learning Process

As previously noted, one of the objectives of this study was to determine if all the information collected through the application of the questionnaire was organized in some way in different CE-TL profiles. To answer this point, a series of cluster analysis of K means was carried out, considering different grouping possibilities. Finally, we opted for a classification in four groups, as detailed in Table 1, highlighting in different shades of gray the election trends in each case.

**TABLE 1:** Conglomerates resulting from the classification with the answers in Scale 1.

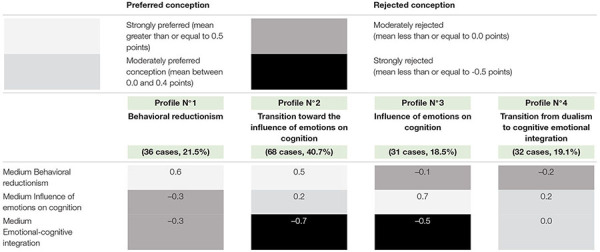

(a) Profile 1 “Behavioral reductionism: the absence of emotions in teaching-learning” (36 cases; 21.5%): This profile can be considered as the most basic of all emerging cluster analyses, due to the orientation it presents toward conception 1 “Behavioral reductionism: the absence of emotions in teaching-learning.” This conception is characterized by ignoring the existence of affections in educational processes by reducing them to their associated behaviors (Bächler and Pozo, 2016). In addition, in a way that is consistent with the preference expressed by the simplest conception, this profile equally rejects the more complex conceptions “Influence of emotions on cognition” and “Emotional-cognitive integration.”

(b) Profile 2: Transition toward the conception of emotions as a context of the teaching-learning processes (68 cases 40.7%): This is the profile that groups the largest number of participants in the study. It presents some similarities in relation to the preceding group because it is also characterized by preferring the conception “Behavioral reductionism.” However, in this case, the preference for behavioral reductionism is shared with the conception “Influence of emotions on cognition,” although with a lower degree of predilection. In a logical sense, preference for the first two conceptions is complemented by the greatest rejection expressed by the most complex conception, called “emotional-cognitive integration.”

(c) Profile 3: “Influence of emotions on cognition: emotions as a context of the teaching-learning process” (31 cases 18.5%): This profile is characterized by preferring practically exclusively conception 2 “Influence of emotions on cognition.” Consistent with the above, this profile presents a neutral position due to the conception 1 “Behavioral reductionism,” as well as an explicit rejection of the more sophisticated conception, “Cognitive emotional integration.” The above implies that students who are grouped under this profile better understand the mental status of the affections; however, they consider the role of emotions on the teaching-learning processes only from the valence of the latter. In other words, if you experience pleasure, you learn more and if you feel discomfort, you learn less (Bächler and Pozo, 2016).

(d) Profile 4 “Transition from dualism to cognitive emotional integration” (32 cases 19.1%): This profile shows a moderate preference for conception 2 “Influence of emotions on cognition.” However, we consider this perspective as a transition point toward emotional-cognitive integration because it is the only profile that parallely expresses a neutral position toward this last conception. This would imply that in certain circumstances the participants associated with this profile, consider emotions as the center of the teaching-learning processes, valuing the role of affections beyond their valence (Bächler and Pozo, 2016).

### Second Part Results: Identification of Emotionally Significant Memories Associated With the School Biography That Preservice Teachers Possess

Considering the findings obtained from the cluster analysis performed, we selected thirty participants with whom we carried out, a process of recovering emotionally significant experiences through imagery that they had lived as students at the school system. Of these thirty participants, fifteen corresponded to the “Behavioral reductionism” profile and fifteen to the “Transition from dualism to cognitive emotional integration” profile. Once the imagery was completed, twenty-five stories were selected for analysis, eliminating five of the original total, in view of different difficulties that prevented the imagery from being carried out in the best way possible.

As noted above, the rescued memories were analyzed following a mixed categorization process (deductive and inductive). First, the corpus was deductively analyzed from *a priori* established two categories, called “positive valence emotional situations” and “negative valence emotional situations.” Then, inductively, an analysis of the memories was carried out, seeking to identify the elements the students described as causes of the different emotions involved in educational situations. As a result of this analysis, five subcategories called “Teacher figure,” “Learning contents,” “Classmates,” “School characteristics,” and “Evaluation” were identified. Finally, also inductively, a “zoom” was carried out on the subcategory “Teacher figure” since this was the cause of emotions, both positive and negative, that appeared more frequently among the memories. As a result of this last analysis, three dimensions emerged of the figure of the teacher as a cause of positive valence emotions (Familiarity’s relationship between teacher and student, Demonstrations of empathy and Positive reinforcement) and four dimensions of the figure of the teacher as a cause of negative valence emotions (Abusive exercise of power, Specific behaviors and traits of teachers, Emotional reliefs of teachers, Disqualifications of student’s cognitive abilities).

Below are two types of results. The first one corresponds to the memories that are associated with emotions of positive valence; and the second refer to stories linked to emotions of negative valence. For both results, the same presentation structure is used which contains the following elements: Initially, the detail of the different emotions identified is shown, illustrating some of the cases with excerpts from the discourses obtained. Subsequently, the different aspects indicated by the participants are analyzed as causes of the emotions they relate to. Finally, a more specific analysis of different aspects associated with teachers is presented, given that the analysis carried out indicates that they would be the main factor of emotions among students.

#### Positive Valence Emotions Identified

**FIGURE 4:**
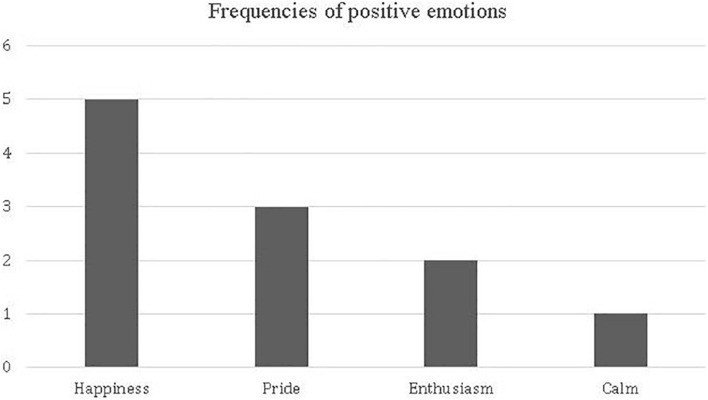
Frequency of emotions of positive valence.

Students revealed having remembered four types of emotions of positive valence during their passage through primary education: happiness, pride, enthusiasm, and calmness. Figure 4 summarizes the frequency of each of these emotions.

When comparing the frequencies of these emotions through the Wilcoxon statistic, we found no differences among them. The following excerpt allows us to exemplify the type of emotionality of positive valence referred to by students when remembering passage through the primary school system:

“My memory was in a language class while the teacher tried to teach us, and I didn’t pay much attention until he said it was for Mother’s Day and that we would recite it in front of them. This motivated me in that language class to write a poem with the number of verses that the teacher asked us, in addition the teacher noticed my enthusiasm and helped me with my poem. After that, he selected my poem to recite on Mother’s Day. This impacted me a lot because they didn’t consider me much in that school because I was thought of as untidy” (TS 14)^2^

As can be seen from this story, the student experienced enthusiasm, an emotion of positive valence that translated into a “motor” perform the activity better. In addition, it seems that in this case, the teacher played a key role in terms of facilitating the emotions that were reported. Below, we analyze this and other causes that students refer to with regard their emotions.

##### Causes of student emotions

**FIGURE 5:**
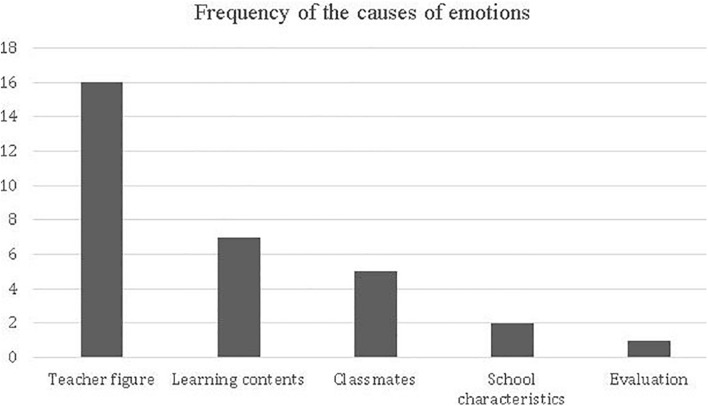
Frequency of the causes of emotions.

When students remembered teaching-learning situations during which they experienced emotions, they identified five different causes for these states: Teacher figure, Learning content, Classmates, School characteristics and emotions associated to the evaluation. These factors influenced the emergence of different types of emotions, both positive and negative. Figure 5 summarizes the frequency of these causes.

When comparing the frequencies of the causes that students identified for the emergence of emotions through the Wilcoxon statistic, we found that teacher is the most recurrent factor among student accounts (Teacher figure v/s learning contents: *Z* = −2.183, *p* = 0.0029; Teacher figure v/s classmates: *Z* = −3.051, *p* = 0.002; Teacher figure v/s school: *Z* = −3.300, *p* = 0.001; Teacher figure v/s evaluation: *Z* = −3.638, *p* = 0.001)]. Regarding the rest of the causes, no differences were observed between them, so that it can be assumed that these factors were of equal importance.

The following excerpt illustrates the importance of teachers as a factor of positive valence emotions experienced by students:

“The classroom was a pleasant place because there was a teacher who was very dear to all, taught language and mathematics (.) my teacher gave books to students who obtained better grades. These had written dedications in them, but the most special thing of all was that there were several of them from which one could choose. She gave us an incentive for everything; or at least I felt that way, to paint, read, draw and explore our world, as well as dance and sing.” (TS 11)

Since we found that teachers are the most relevant factor for the emergence of emotions among students, we decided to perform an analysis by disaggregating the different dimensions of the role of the teacher that is linked to emotions of positive valence, as discussed below.

##### Factors associated with teachers who are identified as the cause of positive emotions

**FIGURE 6:**
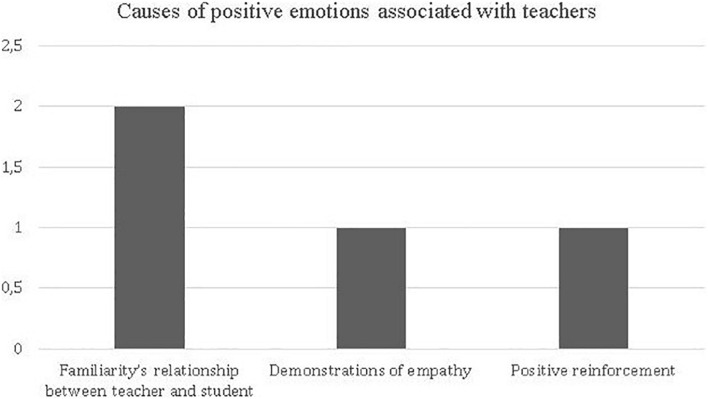
Frequency of different dimensions of the teacher as a facilitator of positive valence emotions, according to the memories of the future teachers analyzed.

To deepen the understanding of traits actions of teachers that students identify as factors of positive valence emotions, we performed a disaggregated analysis of these variables. We identified the following aspects: a Familiarity relationship between teacher and student, Demonstration of empathy and the use of Positive reinforcement. Figure 6 presents the frequency of each of these aspects.

When analyzing the different aspects associated with teachers that provoked positive emotions among students, there were no significant differences that indicated that any of these were decisive for the emergence of these emotions. Below, we illustrate through extracts of memories the first two factors for analysis.

On the familiarity’s relationship between teacher and student:

“For me, math was exceedingly difficult, and in one situation my teacher decided to stay after work to explain or teach me in a better way. I remember we were in the staff room, where there was a giant table and many chairs. [.] what impacted me most in this situation was that she told me she had faith in me and in my ability to learn. I was a girl, and I did not care about these words. He also told me that I should put more effort into it and not feel frustrated about not understanding something, because not everyone had the same abilities. No doubt those words today have been helpful and have helped me get where I am.” (TS 23).

As can be deduced from this excerpt, the student refers to the teacher as a person who goes beyond the role of an instructor, establishing a close relationship with the student. This relationship implies exceeding the established work schedules, in addition to expressing, positively, conceptions regarding the potential of the student. This is a familiarity bond that involves the creation of a space for the expression of emotions. In this sense, familiarity is inseparable from the demonstration of empathy, the second emotion factor we detected; illustrated in the extract below:

“I remember an exceedingly difficult moment that happened when I was in eighth grade and was experiencing a lot of conflicts at home, (…) At the end of a class block the teacher came to talk to me and I decided to tell her about my situation. It is exceedingly difficult for me to express my feelings, but the teacher created a pleasant and conducive environment for me to express myself. She listened to me carefully and gave me the support that was necessary, and the technical tools of the discipline she exercised. I went through an application process to enter secondary school and I was in the top ten of the ranking; and I was also the best student of my course and much of it was thanks to the help and support of that teacher.” (TS 28).

As observed in this extract, demonstrations of empathy are associated with the generation of a pleasant atmosphere and a willingness by the teacher to listen attentively. Both aspects allow for the expression of the emotions of the student, a situation that as it appears from the story, was crucial to the student’s further positive development and learning.

Unfortunately, not all recovered memories are associated with these kinds of pleasant traits and emotions. The imageries also allowed us to recover a series of memories of emotions of negative valence, as discussed below.

#### Negative Emotions Identified

**FIGURE 7:**
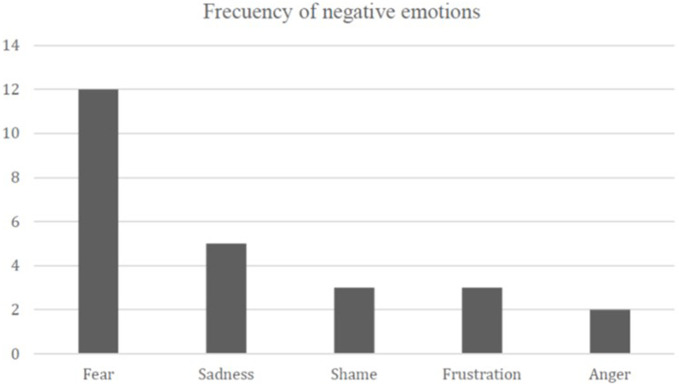
Frequency of negative emotions.

Our analysis shows the existence of five different types of emotions of negative valence that students experienced during their time in primary education: fear, sadness, shame, frustration, and anger. Figure 7 shows the frequency of each of these emotions.

By contrast, through the Wilcoxon statistic method, we found that fear was the most recurring emotion among students’ stories (fear v/s sadness: *Z* = −2.333, *p* = 0.0029; fear v/s shame: *Z* = −2.496, *p* = 0.013; fear v/s frustration: *Z* = 2,496, *p* = 0.013; and fear v/s anger: *Z* = −3,638, *p* = 0.001).

Unfortunately, the results of our study suggest that fear continues to be an important driving factor of educational processes in our school system, as reflected in the following fragment of a student’s memory:

“The room was completely silent with everyone properly seated, only the loud voice of the teacher was heard, and I could only observe my classmates because of the fright we had at that time.” (TS 5).

Within the different aspects that students refer to as causes of the emotions they experienced in the classroom, the role of the teacher stands out. For this reason, as we did in the case of emotions of positive valence, we performed an analysis separating the different dimensions of the role of the teacher that associates the emergence of emotions of negative valence.

##### Factors associated with teachers who are identified as the cause of negative valence emotions

**FIGURE 8:**
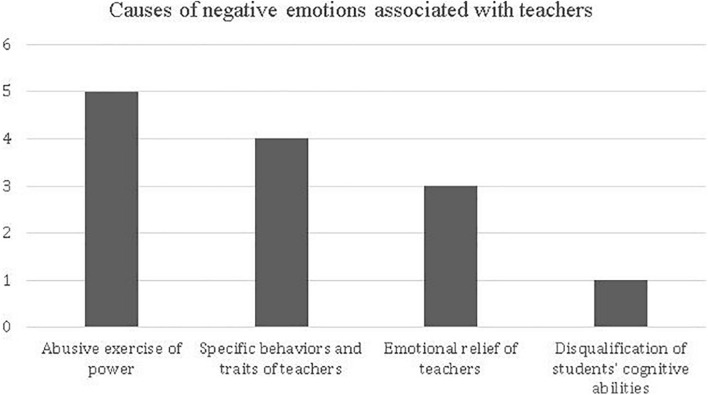
Frequency of the different dimensions of the teacher as a facilitator of negative emotions, according to the memories of the future teachers analyzed.

Participants identified four dimensions associated with the role of the teachers as factors causing emotions of negative valence: Abusive exercise of power; Specific behaviors and traits of teachers, Teachers’ emotional relief, and Disqualification of students’ cognitive abilities. Figure 8 shows the frequency of each of these aspects.

When contrasting each of these aspects using the Wilcoxon statistic method, no differences can be seen. Therefore, we assume that these are aspects that have the same weight as factors to producing emotions of negative valence associated with teachers. The following excerpts from memories illustrate the characteristics of each factor.

###### Abusive exercise of power

“I remembered the math workshop class (…) I was in 3rd grade. (.) The teacher filled the board completely with different types of exercises and explained one of everything and gave us the option that the student who finished could leave the class. My thoughts were of anguish and despair because one of the subjects that was always hard for me was mathematics, and it was neither the first nor the last time I stayed in the room. I would see my sister, angry and desperate. We lived in a rural place, and because of this we would miss our bus every Friday, but the teacher didn’t care.” (TS 21).

As can be seen in the story, there is an asymmetry of power in the teacher-student relationship, which, in this case, the teacher uses in an abusive manner without considering the harm it entailed for the student.

###### Specific behaviors and traits of teachers

The following excerpts, corresponding to two different students, reflect the presence of this factor:

“I remember that I was in an English class with a new teacher, who had come to school, I was afraid of her at the beginning because she spoke very loudly and was very serious.” (TS 13).

“I was able to recall a Spanish class, just because the teacher had an unpleasant, squeaky tone of voice […] she asked me to write a particular phrase from the tree and I wrote on the board about the tree and I remember it, because of that error, she got up from her chair upset, erased what I had written and [called] me to write it properly. She did not correct my mistake, and then with her squeaky voice, she scolded us, [.] I never liked her; she was very hurtful when correcting my classmates making them very vulnerable […] it was not easy for a child to stand in front, and do something wrong, much less if the teacher does not give you the opportunity to correct yourself.” (TS 20).

In the two previous cases, the effect is seen of teacher’s voice on the emotional experience of the students. This characteristic is accompanied, in the second case, by behaviors that are “hurtful” for the student when the teacher “corrects” their work or performance.

It is likely that this type of behavior derives from a certain degree of discomfort experienced by the teacher when students are wrong. If so, the expression of the teacher can be understood as an affective catharsis rather than a genuine attempt to correct the error of her students to promote their learning. The following excerpt gives an account of these types of situations that we have called “emotional relief.”

###### Emotional relief of teachers

“My memory was when I was in second grade (…) I was in math classes and the teacher chose about six or seven students to go to the board to do exercises, we were just learning to subtract with remnants, when it was my turn, I felt nervous, I didn’t know how to do it (.) the teacher got angry because I was just looking, just looking at the numbers. She spoke to me loudly and with an angry face (…) I remember that I did it, I finished it, but with a lot of fear and crying, it was the first and last time I cried in a room in front of the teacher.” (TS 12).

In the previous case, the teacher is bothered by how long the student takes to look at the exercise before solving it. It would seem like an unfair discharge of emotions that, as the student remembers it, is disproportionate to the situation. Therefore, we have called these types of situations as “emotional reliefs.” By using this term, we seek to highlight the fact that in these cases the situation would serve as an “excuse” for the catharsis of emotions that have their origin in other reasons. In this sense, it is likely that the behavior of these teachers is mainly motivated by their emotional state, which is why they manifest themselves with different forms of expression in each case. In some of them, such relief could take the form of disqualification of the students, as can be seen in the following excerpt.

###### Disqualification of students’ cognitive abilities

“During this instance I remember that the teacher judged me because I was not going at the same pace as my classmates. Based on this fact, he began to question me how skilled I was and consequently I began to get frustrated. That was an experience that as a child I hoped to never experience again.” (TS 2).

This experience suggests (as the statistical analysis of the frequencies shows) that there would be no aspects associated with the role of the teacher that are more important as factors of emotions of negative valence. What is behind each factor might be an unpleasant emotion experienced by the student or the teacher, which would be expressed in different ways in the relationship with the students. Supporting this thesis, the memory presented below represents the interaction of several factors in a single person in a teaching-learning situation.

What I remember from first or second grade, a teacher named Claudio was teaching us the addition and subtraction with remnants. […] One day, my classmates, about 15 students, counting me, were more unruly than usual and the teacher got truly angry, turned red and started shouting at us (emotional reliefs), “That we couldn’t understand anything, that our level was extremely low! (Disqualification of the cognitive abilities of the students)” and hit the table causing a great noise (behaviors and specific features of teachers), at that moment I remember that I was very afraid and wanted to cry, I thought that the next thing he hit could be my face [.], I never understood why he got so angry. After a while he calmed down, after leaving the room for five minutes, he came back and said that he would start making surprise checks of addition and subtraction with remnants, starting that same day; there were about one hundred exercises, eighty of sums of two or three digits and twenty subtractions of two and one digit (abusive exercise of power). (TS 19).

Regarding the latter case, the student ends her story by pointing out:

“I realized that all the fear of the teacher that situation had caused me I transferred it to the subject and every time I sat down doing a math exercise, I became pale, I started to sweat, and I was in such anguish that I became so nervous that I couldn’t do them.” (TS 19).

From this imagery, the student becomes aware of how her fear of mathematics is, at least in part, shaped by this painful experience. It is a memory that “impacted” for her entire schooling career, and that most likely, will also shape her conceptions about the educational process. The existence of this eventual link between the recovered memories and the conceptions that the students have about the role of emotions in the teaching-learning processes is examined below, in the last group of results of this study.

### Third Part Results: Identification of Relations Between the Teachers’ Conceptions About the Relationships Between Emotions and Teaching and Learning Processes and the Memories Recovered

**FIGURE 9:**
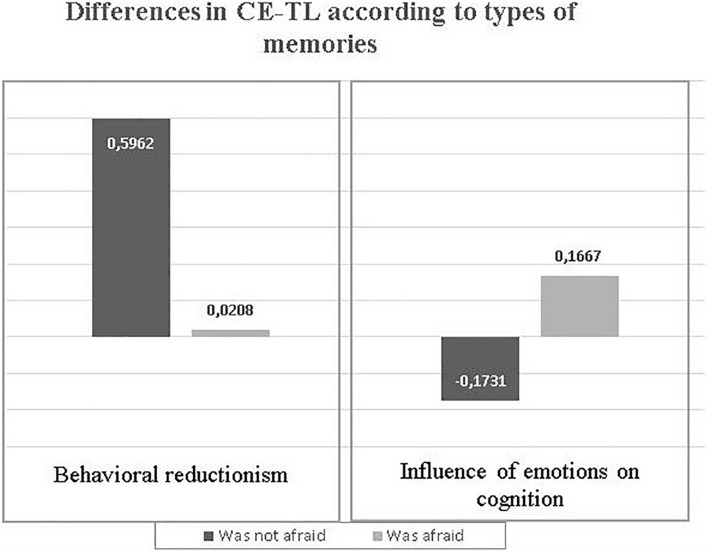
Differences in Teachers’ Conceptions about the relationships between Emotions and Teaching and Learning processes (CE-TL) according to types of memories.

Finally, we carried out an analysis aimed at assessing the possibility that there are different types of conceptions about the relationships between emotions and the teaching-learning processes according to the characteristics of the memories recovered. Figure 9 summarizes these results.

Analysis showed that there were differences in the CE-TL that students had depending on whether they experienced the emotion of fear during primary school. As can be seen in Figure 9, students who did not remember experiencing fear have a degree of acceptance for the conception “Behavioral reductionism,” significantly higher than that expressed by those who remember feeling fear: [*t*(23) = 2,605, *p* = 0.016]. On the other hand, students who remembered experiencing fear during their passage through primary education, expressed a greater acceptance of C2: Influence of emotions on cognition, compared to those who did not indicate having experienced this emotion: [*t*(23) = 2,904, *p* = 0.008].

In summary, the data collected indicate that the experiences of fear experienced during primary education could be a factor that relates to different types of conceptions about the relationships between emotions and teaching-learning processes.

## Discussion

The discussion is organized in four different sections. Firstly, some reflections are presented, which are constructed from the results obtained through the initial part of the study (CE-TL evaluation). Next, the conclusions obtained from the second part of the research are presented (analysis of the emotionally significant school memories). Subsequently, some assessments are made regarding the third category of research results corresponding to the integration between the results of the first and second part of the study. Finally, some reflections about the use of imagery to rescue memories of emotionally significant experiences are presented.

### Reflections About Teachers’ Conceptions About the Relationships Between Emotions and Teaching and Learning Processes Evaluation

As we have already pointed out, there is a shared acceptance among the participants in the study for the simplest conceptions considered in the investigation: “Behavioral reductionism” and “Influence of emotions on cognition.” This acceptance is complemented by the overwhelming rejection expressed by future teachers regarding the more complex conception called “Emotional-cognitive integration.” In fact, none of the profiles identified in the study proved to be fully integrative of emotions in cognitive processes as we found in a study conducted with teachers in practice (Bächler and Pozo, 2020). Given its intrinsic characteristics, the most complex profile that emerged from this research, was considered as “Transition toward emotional-cognitive integration,” in the sense that it would be a profile of students who would be “about to take the leap” toward the most complex conception: “Emotional-cognitive integration.”

What factors, situations, learning and/or other elements will be necessary to overcome this transition? How could this topic be considered in initial teacher training? It is necessary in future studies relate the findings from this research with the characteristics of ITT in emotions (Bächler et al., 2020).

### Reflections About Emotionally Significant School Memories Identified

It can be anticipated, depending on the results of the second part of the present study, that, probably, an ITT in emotions should consider work with the school biographies and the construction of teacher identity of the preservice teachers (Kelchtermans and Deketelaere, 2016), as the results of the second part of our study suggest. Future teachers remember both experiences of positive and negative emotions from their primary education. This is a relevant fact since there are places from which the school is usually conceived as an intrinsically boring and scarcely pleasant space. In these contexts, thinking about the emotions of positive valence that the students were able to experience in school, this type of experience is often associated with games between classmates on the playground or other similar situations not linked to teaching-learning processes in the classroom. However, this is not the case. Participants in this study remember experiencing calm, pride, enthusiasm, and above all joy, learning verbal content such as language, mathematics, and other subjects in the classroom.

How could we enhance this type of experiences in school by transforming them into the *status quo* of education? It is likely that the key to change is found in teachers, since it is, they who are identified as main drivers of emotions in the classroom, both positive and negative. There are teachers who “impact” their students with their demonstrations of empathy, the use of positive reinforcement and the maintenance of closeness or familiarity with their students. Sadly, there are also teachers who, due to certain personality traits and specific behavior, exhibit abusive use of their power in the classroom, disqualification, and what we call in this study emotional relief, leaving a trace of pain and suffering in their students. What will the reasons be behind these unfortunate practices? It is highly likely that it is a problem of multiple origins. Perhaps the precarious working conditions of teachers are related to this problem or, perhaps, it is the high number of students in the classroom. We can say nothing “for sure” about this problem since it was not the focus of the present study. What we can say about it is that, if there is no conscious work with the memories that preservice teachers “drag” from their school history, there is some probability that future teachers will replicate unwanted practices in the future. For example, many recovered memories denote expressions of anger on the part of the teachers in front of the mistakes of their students. What is learned as a student when the teacher is bothered by our mistakes? Probably, these experiences are related to the development of conceptions about error as a problem and not as an opportunity for learning.

### Reflections About the Integration Between the Results of the First and Second Part

The situation described in the previous section, leads us to reflect on the relationship between the memories that the participants in this study recovered and the conceptions they have about the relationships between emotions and teaching-learning processes.

As noted earlier, we obtained a difference in means in the CE-TL that students had as they did or did not recall fear experiences during primary school. Specifically, students who remembered experiencing fear turned out to be less close to “behavioral reductionism” and closer to the conception “Influence of emotions on cognition” than those who did not recover memories of these characteristics.

How can the previous findings be interpreted? These are isolated data that require new studies to be carried out with larger samples and variable control conditions for eventual corroboration. However, beyond the above restrictions, it should also be noted that it is a fact of interest since it could reaffirm the theoretical basis of this study, in the sense of denoting the existence of a relationship between the memories or implicit representations of preservice teachers and their conceptions about the role of emotions in the teaching-learning process. If this interpretation were correct, we draw links between the more and less implicit levels of CE-TL. Perhaps, implicitly, and unconsciously those who experienced fear at school try to avoid replicating painful experiences with their students in the future. This relationship does not, however, reach the most complex conception called “Emotional-cognitive integration,” which remains poorly considered among future teachers. In other words, experiences of fear at school can lead preservice teachers to seek joy, enthusiasm, and other emotions of positive valence as exclusive driving agents of teaching and learning, without considering a true integration of affections in the educational process.

The previous situation runs the risk of becoming the “other side” of the problem, consisting denial of the fact that every educational process is intrinsically emotional, beyond the valence of the affections experienced. It could happen that new generations of teachers seek the promotion of “happy classrooms,” not so much for an understanding of the deep integration between emotions and cognitive processes, but rather due to an attempt not to replicate personally experienced painful situations. Perhaps, part of the emotional rollercoaster that education is currently experiencing has some of this pernicious denial, as some critical authors of positive psychology in the classroom warn (Stout, 2000).

### Reflections About the Use of Imagery to Rescue Memories of Emotionally Significant Experiences

When we consulted the participants in this study if they believed or not that this technique allowed them to access memories that were otherwise more difficult to recover, practically all students answered affirmatively, expressing, in addition, their surprise at the memories obtained. At the same time, the analysis of the stories allowed us to verify that imagery facilitated the recovery of vivid and emotionally charged images, which invites us to think of this technique as one that facilitates access to more implicit levels of some elements of their conceptions regarding other techniques that operate exclusively through verbal language, such as interviews or questionnaires, for example. This invites reflection on the validity and reliability of the imagery as a tool for memory recovery and its possible advantages over other types of instruments. This is a relevant point, among other reasons, since there is a controversy in the literature regarding the ability of imagery to rescue memories objectively, in other words, which refer to events that indeed happened instead of false memories (Arbuthnott, 2005). This can be a deficiency of imagery, for example, when recollection is used as evidence of a criminal act, as in the case of child sexual abuse (Loftus, 1997). Nevertheless, in case of the future teachers’ autobiographical memories, the objectivity of recollection cannot be a problem in terms of its analysis as a constituent element of educational conceptions or conceptions. The main thing to consider, it is the important that their built images have as guides for behavior and decision-making in the classroom (Elbaz, 1983; Goodman, 1988), beyond if these are reliable representations of facts occurred during school history or are subjective construction developed as self-representations about what happened. According to the perspective adopted in this study, an especially important aspect in relation to the recovery of school memories and the technique to be used to achieve this objective, corresponds to the possibility of accessing the subjective nature of the recollection. This idea implies the use of a technique that makes it possible to recover memories that are associated with vivid and emotion-laden images since, following Alliaud (2004) what is relevant to the construction of conceptions is not so much what happened at school but how future teachers experienced what happened there.

Finally, it is important to reflect on the relationship between rescued memories and conceptions in a deep sense, beyond just identifying the statistical association. This relationship is probably mediated by the interpretation that the preservice teachers make of their memories and not necessarily by the characteristics that the memories have in an objective way. Therefore, we believe it is necessary to complement this study with future works that examine the interpretation that teachers make of their memories through interviews, among other research techniques.

## Data Availability Statement

The raw data supporting the conclusions of this article will be made available by the authors, without undue reservation.

## Ethics Statement

The study was reviewed and approved by the Comité de Ética de la Universidad de Playa Ancha. The participants provided their written informed consent to participate in this study.

## Author Contributions

RB was the director of the project that funded the research, designed the study carried out in all its aspects, performed the analysis of the results, and wrote the manuscript. RS was a preservice teacher student who carried out her degree thesis in the context of this project. She participated mainly in operational aspects as a researcher in training. Both authors contributed to the article and approved the submitted version.

## Conflict of Interest

The authors declare that the research was conducted in the absence of any commercial or financial relationships that could be construed as a potential conflict of interest.

## Publisher’s Note

All claims expressed in this article are solely those of the authors and do not necessarily represent those of their affiliated organizations, or those of the publisher, the editors and the reviewers. Any product that may be evaluated in this article, or claim that may be made by its manufacturer, is not guaranteed or endorsed by the publisher.

## Footnotes

1 We use the expressions “preservice teachers,” “future teachers,” and “student teachers” as interchangeably terms in this manuscript.

2 Teacher Student 14.

## Abbreviations

CE-TL: teachers’ conceptions about the relationships between emotions and teaching and learning processes
ITT: initial teachers training
TS: teacher student.
